# Supplementary material for: Abrupt changes of hydrothermal activity in a lava dome detected by combined seismic and muon monitoring
Source: Sci Rep. 2019 Feb 28;9:3079. doi: 10.1038/s41598-019-39606-3 (PMC6395750; doi:10.1038/s41598-019-39606-3)
Supplement: Supplementary file 1 — Supplementary information [file 41598_2019_39606_MOESM1_ESM.pdf]

Abrupt changes of hydrothermal activity in a lava dome detected by combined seismic and muon monitoring

Yves Le Gonidec[1], Marina Rosas-Carbajal[2], Jean de Bremond d'Ars[1], Bruno Carlus[3], Jean-Christophe Ianigro[3], Bruno Kergosien[1], Jacques Marteau[3] & Dominique Gibert[1,4]

[1] Univ Rennes, CNRS, Géosciences Rennes - UMR 6118, F-35000 Rennes, France. [2] Institut de Physique du Globe de Paris, CNRS - UMR 7154, F-75005 Paris, France. [3] Univ Claude Bernard, CNRS, Institut de Physique Nucléaire de Lyon - UMR 5822, Lyon, France. [4] National Volcano Observatory Service, CNRS, OSUR - UMS 3343, F-35000 Rennes, France.

The authors may be joined at the following e.mails: yves.legonidec@univ-rennes1.fr; rosas@ipgp.fr; bremond@univ-rennes1.fr; carlus@ipnl.in2p3.fr; ianigro@ipnl.in2p3.fr; bruno.kergosien@univ-rennes1.fr; marteau@ipnl.in2p3.fr; dominique.gibert@univ-rennes1.fr.

SUPPLEMENTARY INFORMATION (SREP-18-29910A)

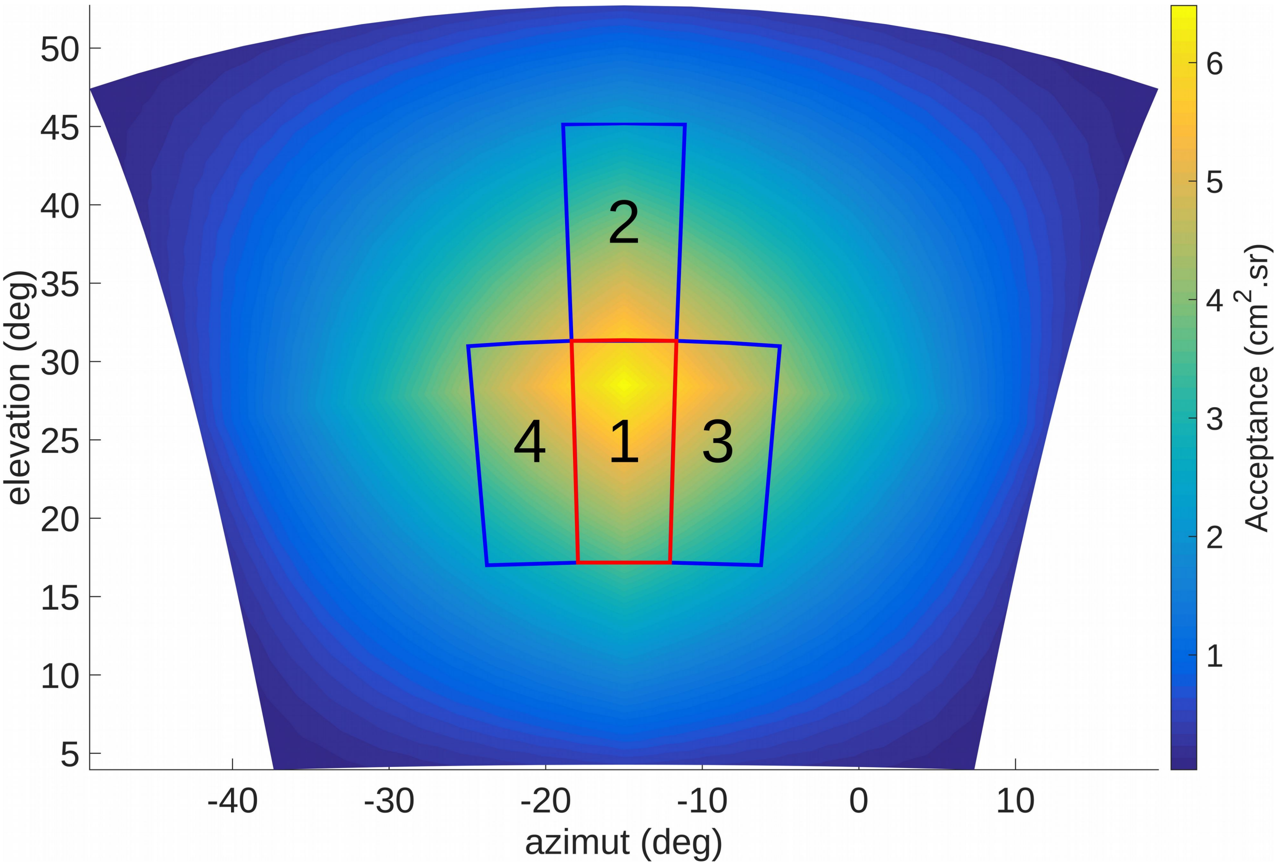

Extended Data Fig. 1 | Telescope acceptance function and merged lines of sight. View field covered by the 19^2 = 361 lines of sight of the cosmic muon telescope. The red rectangle encompass the lines of sight covering the seismic source zone of Fig. 3. These lines are merged to obtain a joined acceptance of 54.3 cm^{2}sr. The time variations of the muons count in this area correspond to the red curve in Fig. 4. The blue rectangles located above and on both sides of area 1 have muons counts shown as blue curves in Fig. 4. The joined acceptances of areas 2, 3 and 4 are respectively: 34.8 cm^{2}sr, 40.3 cm^{2}sr and 45.8 cm^{2}sr. The maximum acceptance of the axial line of sight of the telescope equals 6.5 cm^{2}sr.

Cycle #	Start date (March 2017 UTC)	End date (March 2017 UTC)	Date of maximum (March 2017 UTC)	Duration (h)	Rise time (h)	Fall time (h)	Rise temperature (C)	Fall temperature (C)
**1**	28 17:33	28 20:32	28 19:22	2.98	1.82	1.17	0.12	0.13
**2**	28 20:32	29 00:00	28 22:23	3.47	1.85	1.62	0.13	0.14
**3**	29 00:00	29 04:20	29 02:59	4.33	2.98	1.35	0.25	0.16
**4**	29 04:20	29 09:05	29 07:23	4.75	3.05	1.70	0.20	0.20
**5**	29 09:05	29 14:25	29 12:51	5.33	3.77	1.57	0.36	0.12
**6**	29 14:25	29 19:16	29 17:35	4.85	3.17	1.68	0.15	0.19
**7**	29 19:16	30 03:20	30 00:05	8.07	4.82	3.25	0.32	0.34
**8**	30 03:20	30 12:25	30 10:17	9.08	6.95	2.13	0.41	0.26
**9**	30 12:25	30 20:38	30 18:39	8.22	6.23	1.98	0.47	0.39
**10**	30 20:38	31 05:35	31 02:40	8.95	6.03	2.92	0.65	0.06

Extended Data Table 1 | Data of the temperature cycles observed in the time series of Fig. 2A.

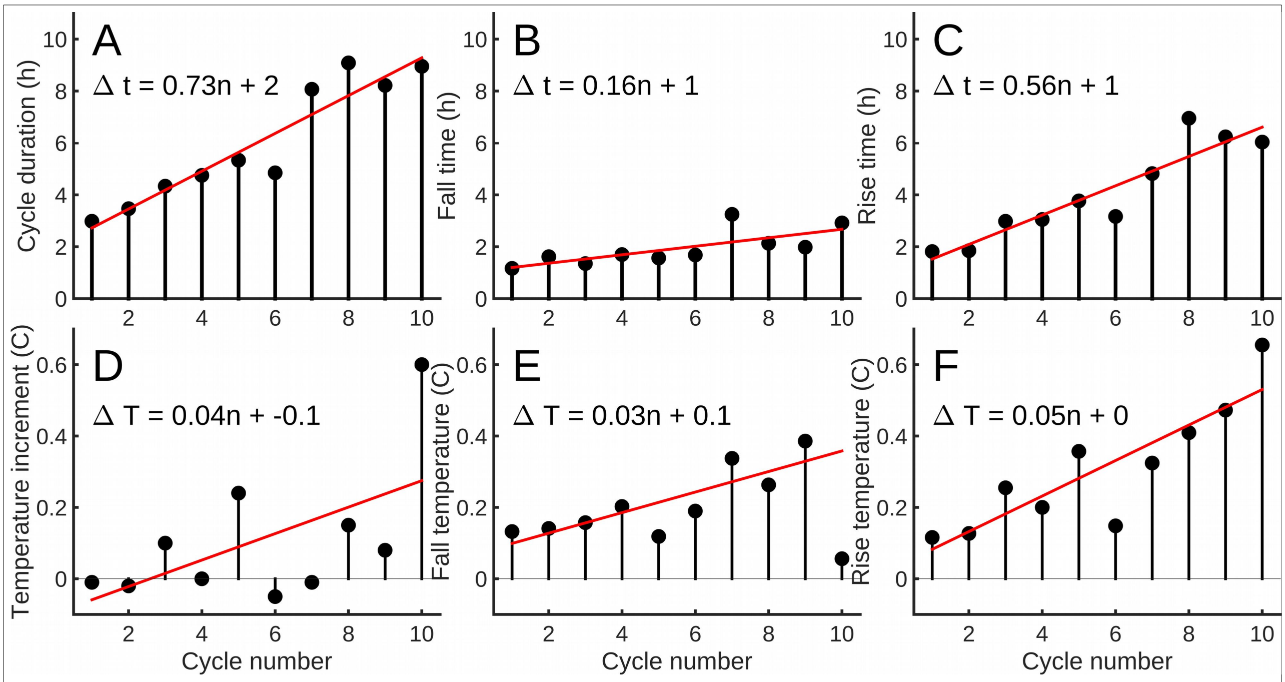

Extended Data Fig. 2 | Main characteristics of the temperature cycles observed in the time series of Fig. 2A. A cycle is defined as period separating two successive minima in the temperature time series of Fig. 2A. By this way, 10 cycles are identified (see Extended Data Table 1 for data values). A: Cycle duration (in hours) defined as the interval between successive temperature minima. B: Temperature fall time (in hours) separating a temperature maximum from the next minimum. C: Temperature rise time (in hours) separating a temperature minimum from the next maximum. D: Increase of temperature between successive minima. E: Temperature decrease between a maximum and the next minimum. F: temperature increase between a minimum and the next maximum. Red straight lines represent the best first-order polynomial fit. Polynomial formula is indicated in each plot. The fall temperature (E) observed during the decreasing phase of the cycles displays a positive trend which, when added with the global positive trend (D), compensates the trend of the rise temperature (F). This indicates that, when corrected for the global trend, a cycle returns to its starting temperature. The temperature time series of Fig. 2A is them the superimposition of a trend and the sequence of oscillations. The temperature trend may be due to a constant steam supply causing a progressive increase of the pressure in the source zone of Fig. 3.

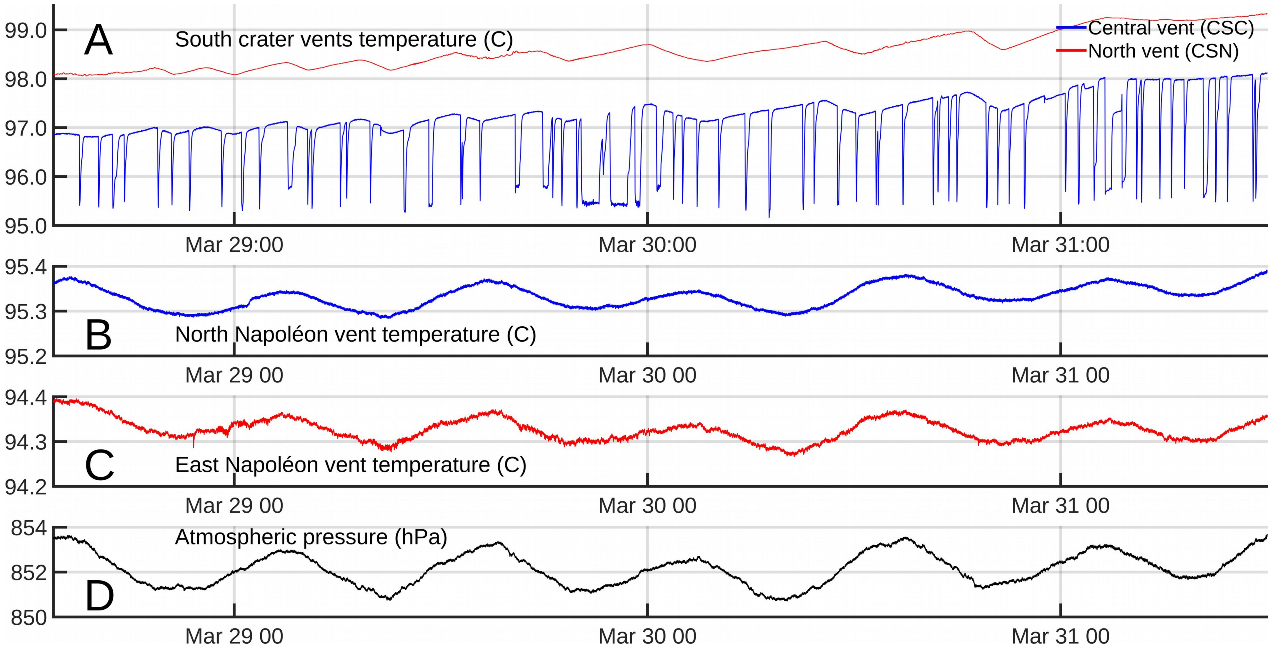

Extended Data Fig. 3 | Atmospheric pressure and Temperature time series in vents. See Fig. 1 for location of the vents. A: Temperature time-variations in the Central and North vents of the South crater. The temperature oscillations observed in the North vent time-series are retrieved in the upper envelope of the Central vent time-series. B: Temperature time-variations in the North Napoléon vent. C: Temperature time-variations in the East Napoléon vent. D: Time-variations of the atmospheric pressure measured at the data concentrator location. Both the North and East Napoléon vents have a low-pressure flux and do not display the remarkable oscillations visible in the vents of the South crater. Instead, the small temperature variations of the Napoléon vents are in phase with the fluctuations of atmospheric pressure.

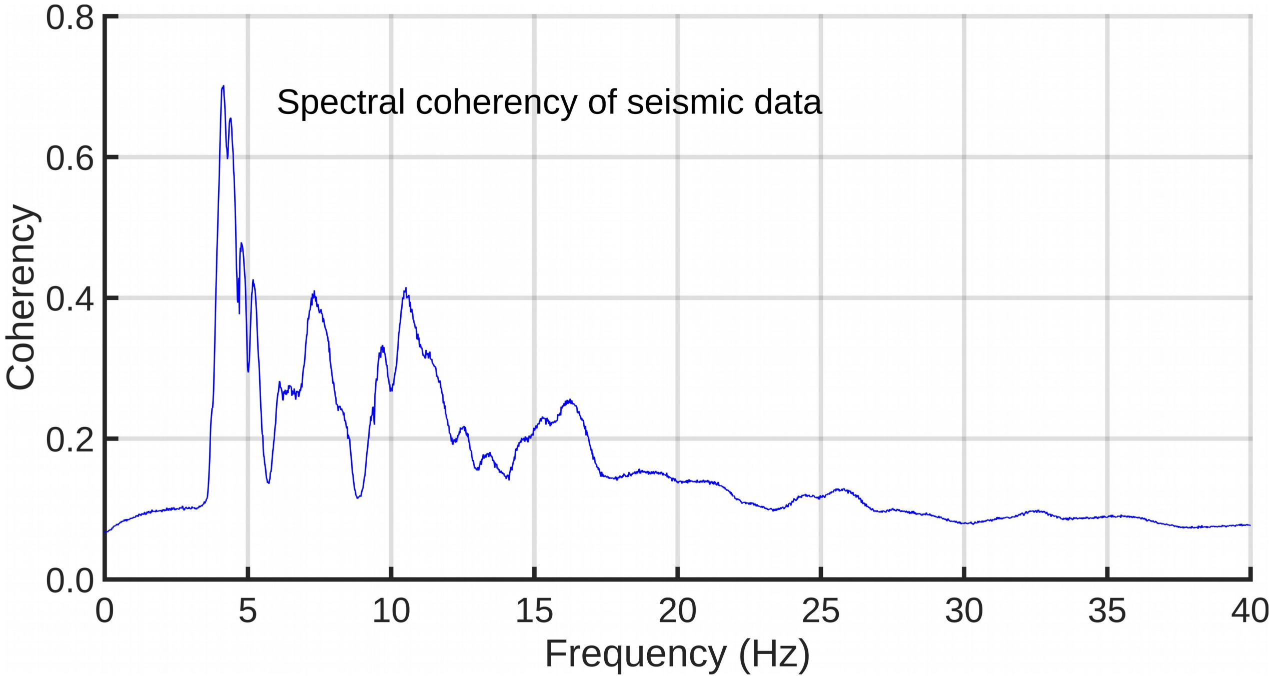

Extended Data Fig. 4 | Spectral coherency of the seismic noise data. The spectral coherency is computed for all seismic data of the POCS antenna according to the method described in Bendat and Piersol[1]. Harmonic spectral lobes are clearly visible.

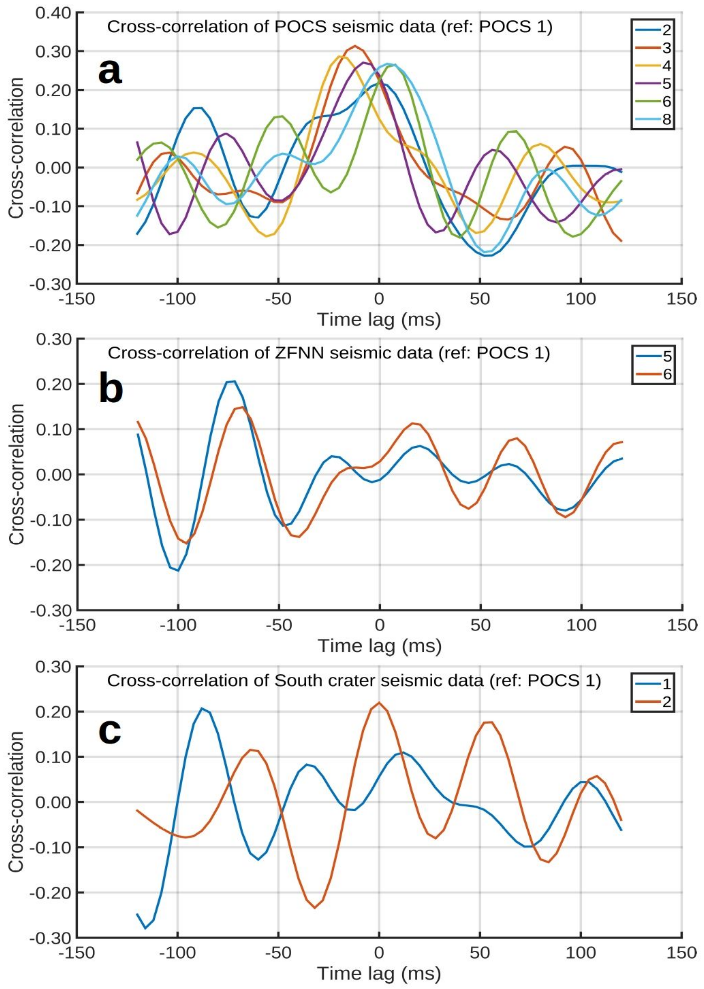

Extended Data Fig. 5 | Cross-correlations of the seismic data. The graphs represent the cross-correlation functions of the geophone data used to localize the source of seismic noise (Fig. 3). All cross-correlations have been computed with respect to geophone 1 of the POCS antenna (Fig. 1). The data are band-pass filtered in the 3-25 Hz frequency band where the spectral coherency is significant (Extended Data Fig. 4). The geophones that are not been used in this study are discarded because of either an electrical problem (POCS #7 and NN #3, 8] or a too low signal-to-noise ratio possibly caused by a poor mechanical coupling with the unconsolidated granular soil and insignificant cross-correlation (SC #3, NN #1, 2, 4, 7).

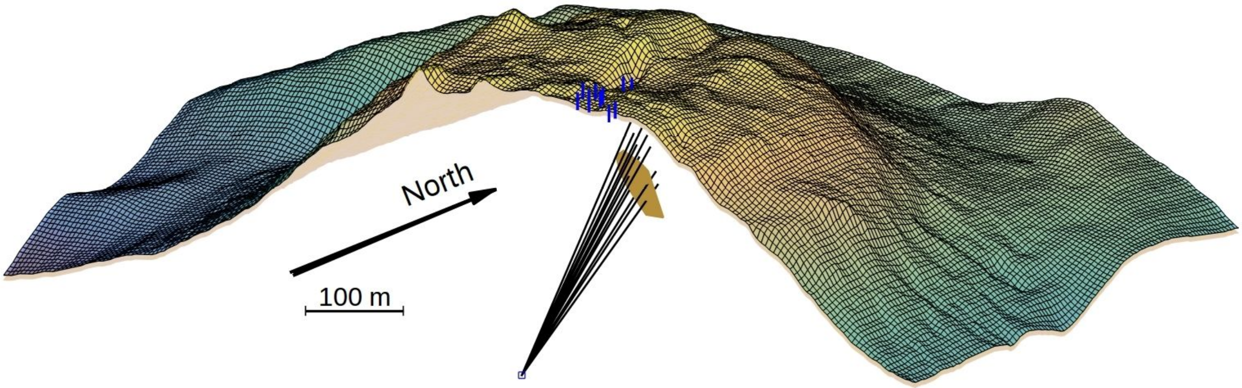

Extended Data Fig. 6 | Location of the active hydrothermal spot in the La Soufrière lava dome. See Fig. 3 for other details.
